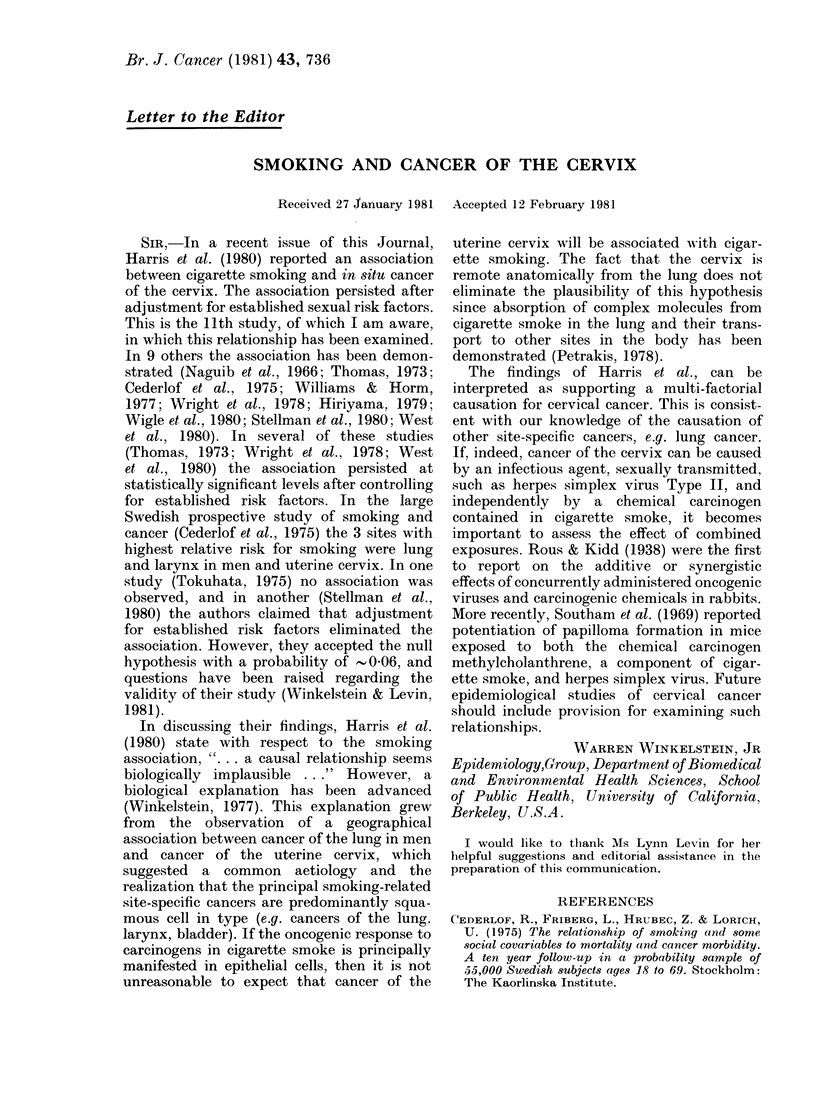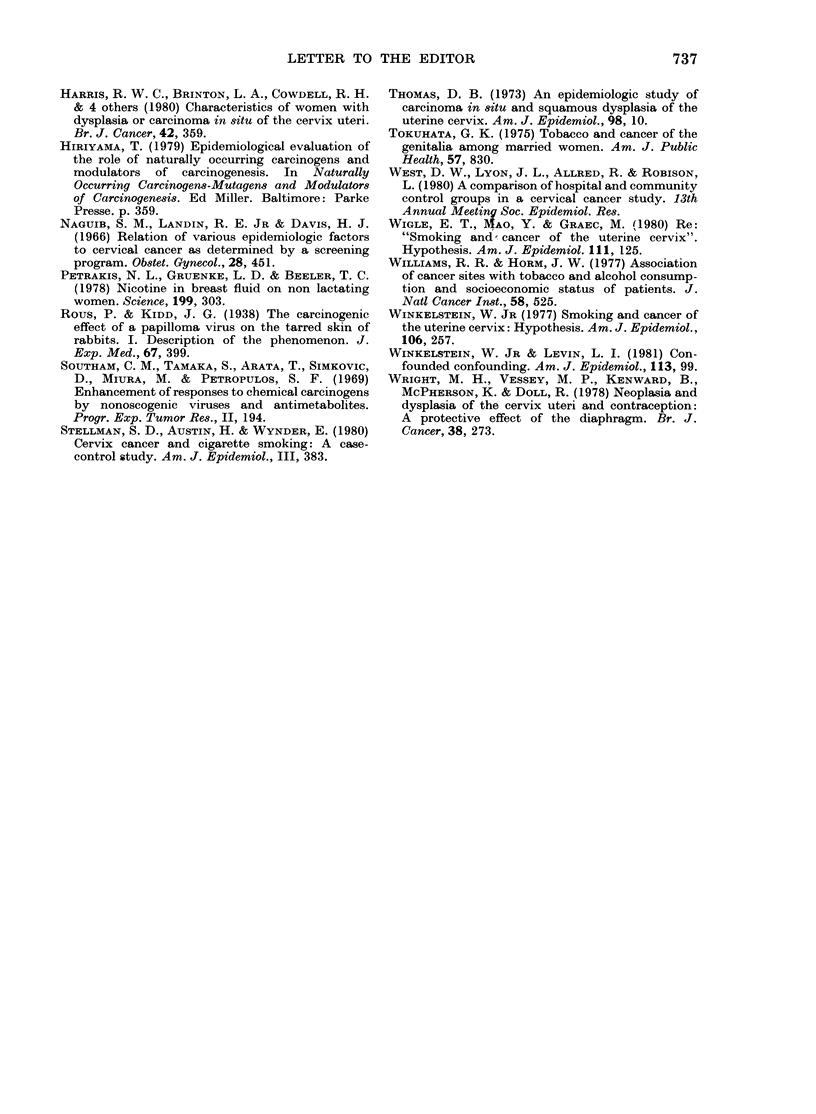# Smoking and cancer of the cervix.

**DOI:** 10.1038/bjc.1981.103

**Published:** 1981-05

**Authors:** W. Winkelstein


*Br. J. Cancer* (1981) **43**, 736

### *Letter to the Editor*

### SMOKING AND CANCER OF THE CERVIX

Received 27 January 1981 Accepted 12 February 1981

SIR,—In a recent issue of this Journal, Harris *et al.* (1980) reported an association between cigarette smoking and *in situ* cancer of the cervix. The association persisted after adjustment for established sexual risk factors. This is the 11th study, of which I am aware, in which this relationship has been examined. In 9 others the association has been demonstrated (Naguib *et al.*, 1966; Thomas, 1973; Cederlof *et al.*, 1975; Williams & Horm, 1977; Wright *et al.*, 1978; Hiriyama, 1979; Wigle *et al.*, 1980; Stellman *et al.*, 1980; West *et al.*, 1980). In several of these studies (Thomas, 1973; Wright *et al.*, 1978; West *et al.*, 1980) the association persisted at statistically significant levels after controlling for established risk factors. In the large Swedish prospective study of smoking and cancer (Cederlof *et al.*, 1975) the 3 sites with highest relative risk for smoking were lung and larynx in men and uterine cervix. In one study (Tokuhata, 1975) no association was observed, and in another (Stellman *et al.*, 1980) the authors claimed that adjustment for established risk factors eliminated the association. However, they accepted the null hypothesis with a probability of $\sim 0{\cdot}06$, and questions have been raised regarding the validity of their study (Winkelstein & Levin, 1981).

In discussing their findings, Harris *et al.* (1980) state with respect to the smoking association, "... a causal relationship seems biologically implausible ..." However, a biological explanation has been advanced (Winkelstein, 1977). This explanation grew from the observation of a geographical association between cancer of the lung in men and cancer of the uterine cervix, which suggested a common aetiology and the realization that the principal smoking-related site-specific cancers are predominantly squamous cell in type (*e.g.* cancers of the lung, larynx, bladder). If the oncogenic response to carcinogens in cigarette smoke is principally manifested in epithelial cells, then it is not unreasonable to expect that cancer of the uterine cervix will be associated with cigarette smoking. The fact that the cervix is remote anatomically from the lung does not eliminate the plausibility of this hypothesis since absorption of complex molecules from cigarette smoke in the lung and their transport to other sites in the body has been demonstrated (Petrakis, 1978).

The findings of Harris *et al.*, can be interpreted as supporting a multi-factorial causation for cervical cancer. This is consistent with our knowledge of the causation of other site-specific cancers, *e.g.* lung cancer. If, indeed, cancer of the cervix can be caused by an infectious agent, sexually transmitted, such as herpes simplex virus Type II, and independently by a chemical carcinogen contained in cigarette smoke, it becomes important to assess the effect of combined exposures. Rous & Kidd (1938) were the first to report on the additive or synergistic effects of concurrently administered oncogenic viruses and carcinogenic chemicals in rabbits. More recently, Southam *et al.* (1969) reported potentiation of papilloma formation in mice exposed to both the chemical carcinogen methylcholanthrene, a component of cigarette smoke, and herpes simplex virus. Future epidemiological studies of cervical cancer should include provision for examining such relationships.

WARREN WINKELSTEIN, JR
*Epidemiology,Group, Department of Biomedical and Environmental Health Sciences, School of Public Health, University of California, Berkeley, U.S.A.*

I would like to thank Ms Lynn Levin for her helpful suggestions and editorial assistance in the preparation of this communication.

### REFERENCES

CEDERLOF, R., FRIBERG, L., HRUBEC, Z. & LORICH, U. (1975) *The relationship of smoking and some social covariables to mortality and cancer morbidity. A ten year follow-up in a probability sample of 55,000 Swedish subjects ages 18 to 69.* Stockholm: The Kaorlinska Institute.

HARRIS, R. W. C., BRINTON, L. A., COWDELL, R. H. & 4 others (1980) Characteristics of women with dysplasia or carcinoma *in situ* of the cervix uteri. *Br. J. Cancer*, **42**, 359.

HIRIYAMA, T. (1979) Epidemiological evaluation of the role of naturally occurring carcinogens and modulators of carcinogenesis. In *Naturally Occurring Carcinogens-Mutagens and Modulators of Carcinogenesis*. Ed Miller. Baltimore: Parke Presse. p. 359.

NAGUIB, S. M., LANDIN, R. E. JR & DAVIS, H. J. (1966) Relation of various epidemiologic factors to cervical cancer as determined by a screening program. *Obstet. Gynecol.*, **28**, 451.

PETRAKIS, N. L., GRUENKE, L. D. & BEELER, T. C. (1978) Nicotine in breast fluid on non lactating women. *Science*, **199**, 303.

ROUS, P. & KIDD, J. G. (1938) The carcinogenic effect of a papilloma virus on the tarred skin of rabbits. I. Description of the phenomenon. *J. Exp. Med.*, **67**, 399.

SOUTHAM, C. M., TAMAKA, S., ARATA, T., SIMKOVIC, D., MIURA, M. & PETROPULOS, S. F. (1969) Enhancement of responses to chemical carcinogens by nonoscogenic viruses and antimetabolites. *Progr. Exp. Tumor Res.*, II, 194.

STELLMAN, S. D., AUSTIN, H. & WYNDER, E. (1980) Cervix cancer and cigarette smoking: A case-control study. *Am. J. Epidemiol.*, III, 383.

THOMAS, D. B. (1973) An epidemiologic study of carcinoma *in situ* and squamous dysplasia of the uterine cervix. *Am. J. Epidemiol.*, **98**, 10.

TOKUHATA, G. K. (1975) Tobacco and cancer of the genitalia among married women. *Am. J. Public Health*, **57**, 830.

WEST, D. W., LYON, J. L., ALLRED, R. & ROBISON, L. (1980) A comparison of hospital and community control groups in a cervical cancer study. *13th Annual Meeting Soc. Epidemiol. Res.*

WIGLE, E. T., MAO, Y. & GRAEC, M. (1980) Re: "Smoking and cancer of the uterine cervix". Hypothesis. *Am. J. Epidemiol.* **111**, 125.

WILLIAMS, R. R. & HORM, J. W. (1977) Association of cancer sites with tobacco and alcohol consumption and socioeconomic status of patients. *J. Natl Cancer Inst.*, **58**, 525.

WINKELSTEIN, W. JR (1977) Smoking and cancer of the uterine cervix: Hypothesis. *Am. J. Epidemiol.*, **106**, 257.

WINKELSTEIN, W. JR & LEVIN, L. I. (1981) Confounded confounding. *Am. J. Epidemiol.*, **113**, 99.

WRIGHT, M. H., VESSEY, M. P., KENWARD, B., MCPHERSON, K. & DOLL, R. (1978) Neoplasia and dysplasia of the cervix uteri and contraception: A protective effect of the diaphragm. *Br. J. Cancer*, **38**, 273.